# Christiaan Eijkman (1856–1930)

**DOI:** 10.1007/s00415-018-9162-7

**Published:** 2018-12-26

**Authors:** Krzysztof Pietrzak

**Affiliations:** The Department of Spondyloorthopedics and Biomechanics of the Spine, University of Medical Sciences Poznań, 28 czerwca 1958 137/147, 61-501 Poznan, Poland


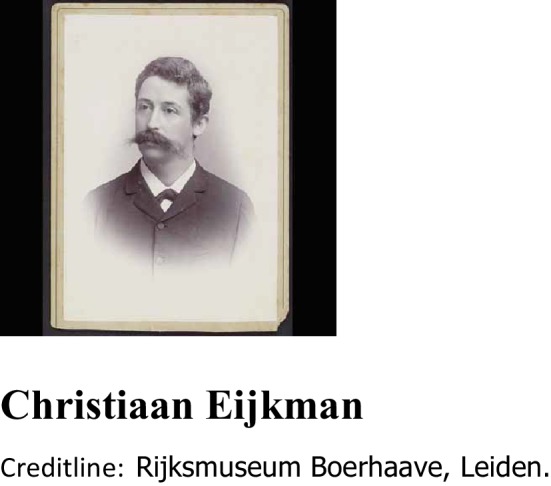


Christiaan Eijkman (1856–1930) was born on 11 August 1856 in Nijkerk, Netherlands. His father, Christiaan, was a school master and his mother, Johanna Alida Pool, took care of their large family (Christian was the seventh child). In 1875 Christian Eijkman enrolled in the University of Amsterdam Military Medical School wishing to later join the Netherlands Indies Army. In 1883 in Amsterdam, Netherlands, he received his doctoral degree with distinction for the thesis On Polarization of the Nerves [1].

Pursuing his vocation in 1883 he left the Netherlands to start work in the Dutch East Indies. In 1885, when in Java, he contracted malaria and was forced to return to Europe to receive treatment. This experience sparked his life-long interest in tropical diseases. He established contacts with a research laboratory in Amsterdam. However, more important was his work for Robert Koch (1843–1910) in Koch’s Berlin laboratory. This is where Eijkman started working with Cornelis Pekelharing (1848–1922) and Tiberius Winkler (1822–1897), who were then preparing for a research expedition to study beri–beri. The disease, with difficulty in walking, tingling or loss of sensation in hands and feet, loss of tendon reflexes muscle function or paralysis of the lower legs and mental confusion, was widespread in the Dutch Indies.

He joined them as a medical officer and returned to Java in 1887. However, Pekelharing and Winkler were soon recalled to the Netherlands. Thanks to their support and Eijkman’s knowledge of research methods Eijkman was not only able to continue the work, but was also appointed head of “Dokter Djava School” in 1887. This marked the end of his career in the army. Eijkman now focused on his research. In 1888–1896 he was also director of the Medical Laboratory, where he dispelled a number of myths on acclimatization of Europeans in the tropics. He proved that blood parameters of newcomers acclimatizing to new conditions did not change and that there were no differences between the physiology of breathing or metabolism between the Europeans and the indigenous population [2].

In 1884, Takaki Kanehiro (1849–1920), a Japanese Navy doctor, eliminated white rice from sailors’ diet and added nutrient-rich food to their diet. This change led to the eradication of beri–beri among sailors during a several-month-long voyage. Takaki mistakenly attributed this to the nitrogen-rich diet. The diet, however, proved to be too expensive and the Navy did not continue with the diet change. Apart from the costs, another contributing factor was a deep-seated belief that the underlying causes of beri–beri were infectious. Consequently, the idea that the disease could be caused by a diet seemed far-fetched. Takaki’s discovery was soon forgotten [3].

The objective of the studies in the Dutch Indies was to find the cause of beri–beri. Eijkman’s first idea was that the disease was caused by bacteria. To investigate this this Eijkman had animals infected with bacteria, but they did not develop the disease. He concluded that longer incubation was necessary. To confirm this he purchased a number of chickens. He got some of them infected while others were not exposed to any pathogen. Surprisingly, all of them fell ill. Eijkman believed that healthy chickens contracted the disease from sick chickens. To prove this he bought another batch and kept them in separate cages. However, the chicken still got sick. This led Eijkman to conclude that the whole laboratory was infected so he placed the chickens in a new place. These chicken also fell ill. Eijkman was bewildered.

His further research was inspired by a fortunate accident. The chickens which were fed polished rice, purchased for the army, had polyneuritis, a disease whose symptoms resemble beri–beri. After a few months the army cook refused to serve such rice to soldiers. Chickens’ diet also changed and they were now fed regular rice, with a silver layer. Following diet change their health rapidly improved. Eijkman noticed the change in chicken fodder and concluded that the two feeds must have differed, with the latter containing an agent that could prevent or cure beri–beri.

Eijkman continued his observations of the subsequent stages of the disease and performed autopsies. He found that the clinical changes in sick chickens were identical to those in beri–beri and described histological lesions typical of polyneuritis endemica perniciosa. He named the condition polineuritis galliniarium. In later studies he proved that the disease was not caused by changes in blood, breathing, temperature or climate. He also ruled out the role of protein and minerals in rice husk in the development of the disease [4, 5]. In 1885 he started working with the head of the health authorities in Java, Adolphe Vordermann (1944–1902), and carried out large-scale studies with the participation of prisoners. A change of diet helped many of them to recover. Out of 95,000 prisoners who ate rough rice only 9 fell ill while the incidence of the disease in a group of 150,000 prisoners who ate polished rice was 4200. This definitively proved that a diet change (in particular the elimination of polished rice) played a role in disease prevention and treatment.

Sadly, Eijkman became a target of attacks, and his superiors pointed out the alleged mistakes made by Eijkman and Vorderman in their studies. This adversely affected the impact of their research within the scientific community. The impact was additionally limited due to the fact that the papers were published in Dutch [5, 6]. What element of the rough rice had a curative effect remained a mystery. Eijkman called it the “anti-beri-beri factor”.

After he fell ill, Eijkman was no longer able to continue his research in the Indies. Other researchers began to suspect that it was not so much that the substance in rice husk is an antidote to the disease but that the deficiency of the substance causes the disease. At Eijkman’s request and under his supervision the research was continued by Adolphe Vorderman and Gerrit Grijns (1865–1944), who put forward an idea that some substances may be essential for a body to function properly [7]. No one, however, had managed to isolate the substance itself.

It was Casimir Funk (1884–1967), who in 1911 finally isolated the substance from rice husk and called it “amine”. Funk put forward a comprehensive theory that there are substances which are essential to life. He called them “vitae animae”, which he later shortened to “vitamine” [8]. The chemical structure of the substance was first characterized by Robert Williams (1886–1965). Later, in 1936 Williams was the first researcher to synthesize thiamine.

Eijkman returned to the Netherlands in 1898. He was appointed Professor in Hygiene and Forensic Medicine at Utrecht. He devoted himself to research on bacteriology, hygiene, and public health. He became famous for inventing a test based on fermentation, called Eijkman’s test, which helps to determine if water was contaminated by human or animal feces and to check for the presence of *E. coli* [9]. He battled against the spread of alcoholism and tuberculosis.

In 1907 he was appointed Member the Dutch Royal Academy of Sciences and a Correspondent of the American Academy of Sciences. He was also awarded a number of state orders. However, the ultimate recognition of his contribution to science was the Nobel Prize in medicine, which he received in 1929 “for his discovery of the antineuritic vitamin” [10].

Eijkman died in Utrecht on 5 November 1930, after a long disease. He was married twice. After his first wife, Aaltje Wigeri van Edema (1858–1886) died, he married Bertha Julie Louise van der Kemp (1869–1940) in 1888. Their son, Peter (1892–1959), also became a doctor.

Many researchers contributed to the discovery of a link between thiamine and neurological conditions. Many of them, including Casimir Funk, were not honoured with the Nobel Prize. This, however, does not make Christiaan Eijkman’s contribution any less significant. His research proving that beri–beri is caused by lack of an agent found in food provided the much-needed breakthrough. He also started the actual treatment of patients suffering from beri–beri. His work was later continued and extended by other outstanding researchers, whose achievements, however, often did not receive due recognition.
